# Electrochemical Cytokine Profiling Reveals Dual Immunomodulatory Effects of Human Milk Oligosaccharides in THP‑1 Macrophages

**DOI:** 10.1021/acsinfecdis.6c00388

**Published:** 2026-09-30

**Authors:** Victoria A. Federico, Hannah A. Richards, Julie A. Talbert, Jocelyn R. Leal, Jennifer A. Gaddy, Steven D. Townsend, David E. Cliffel

**Affiliations:** † Department of Chemistry, 5718 Vanderbilt University, Nashville, Tennessee 37235-1822, United States; ‡ Department of Chemistry, 3776 Haverford College, 370 Lancaster Avenue, Haverford, Pennsylvania 19041, United States; § Department of Medicine, Vanderbilt University Medical Center, Nashville, Tennessee 37232-2625, United States

**Keywords:** human milk oligossaccharides, inflammation, biosensors, electrochemical sensor, immunoassays, magnetic beads

## Abstract

Human milk oligosaccharides (HMOs) are bioactive carbohydrates that govern host–microbe interactions and immune regulation. Beyond their antimicrobial and antibiofilm functions, HMOs have been shown to modulate inflammatory pathways by altering cytokine expression, including interleukin-1β (IL-1β) and tumor necrosis factor-α (TNF-α), which are key mediators in infection and inflammation associated complications. In this study, we characterize the effects of HMOs on cytokine secretion in THP-1 macrophages stimulated with *lipopolysaccharides* using a validated magnetic bead electrochemical sandwich assay (MBESA). The electrochemical assay enabled precise quantification of cytokine responses and revealed that HMO treatment attenuated proinflammatory signaling in LPS stimulated macrophages. These findings provide new insights into the immunomodulatory properties of HMOs and demonstrate the utility of electrochemical biosensing platforms for rapid, sensitive assessment of cellular inflammatory responses. Future studies will expand this approach to microphysiological systems to further elucidate HMO mediated regulation of immune signaling.

## Graphical abstract

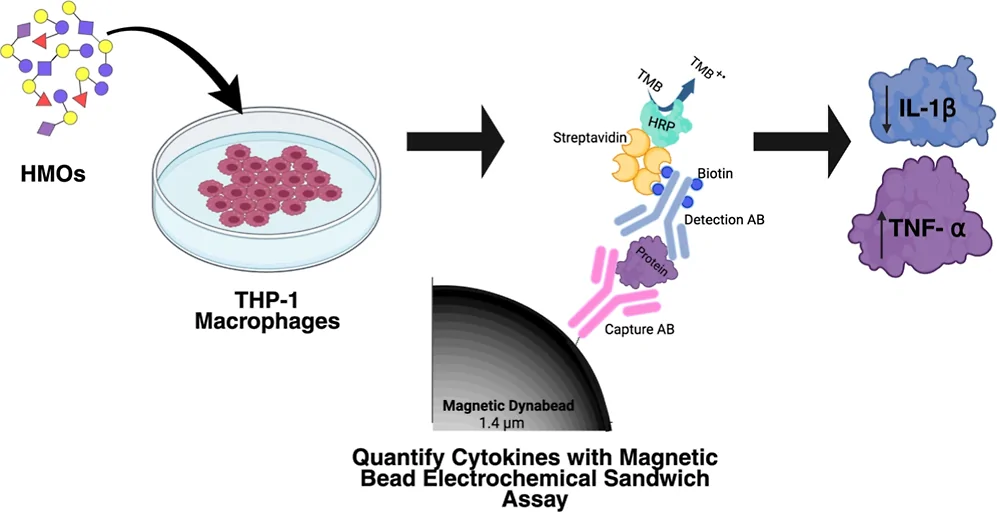

## Introduction

Inflammation is a fundamental component of the body’s immune response, yet when dysregulated, it underlies a broad spectrum of acute and chronic diseases. Proinflammatory cytokines such as interleukin-1β (IL-1β) and tumor necrosis factor-α (TNF-α) serve as key mediators of immune activation and tissue signaling, and their levels are increasingly recognized as important biomarkers for monitoring inflammatory responses. Indeed, elevated inflammatory cytokine concentrations in amniotic or cervicovaginal fluid have been strongly linked to spontaneous preterm birth and perinatal infections, including those caused by Group B *Streptococcus* (GBS) and *Escherichia coli* (*E. coli*), suggesting that local inflammatory signaling at the maternal–fetal interface may trigger early labor.^1^ Similarly, in autoimmune conditions such as rheumatoid arthritis, the expanded production of TNF-α and IL-1β contributes directly to synovial inflammation, joint destruction, and systemic comorbidities.^2^ In the context of gastrointestinal immune disorders like Crohn’s disease^3^ or colorectal cancers,^4^ persistently elevated TNF-α and IL-1β levels correlate with disease activity and mucosal inflammation. Given the central role of these cytokines across diverse inflammatory pathologies, the ability to rapidly monitor their secretion and responses in relevant cellular systems is critical for both mechanistic understanding and therapeutic development. In this context, bioactive molecules such as human milk oligosaccharides (HMOs) have emerged as important modulators of inflammatory signaling,^5^ highlighting the need for integrated analytical approaches to investigate their immunological effects.

HMOs are the third most abundant macromolecule found in human breast milk, following lactose and lipids, with physiologically relevant concentrations ranging from 5 to 25 mg/mL.^5^ Although they are composed of only five monosaccharide building blocks, d-glucose, d-galactose, *N*-acetylglucosamine, l-fucose, and *N*-acetylneuraminic acid (d-sialic acid), more than 200 distinct HMO structures have been identified.^6,7^ HMOs play a vital role in shaping the infant immune system and developing the gut microbiome, primarily by promoting the growth of beneficial gut bacteria while limiting the colonization of pathogens.^32^ Beyond these physiological functions, HMOs also exhibit direct antimicrobial and antibiofilm activities, inhibiting pathogens such as GBS.^5,8^ In addition, HMOs enable dose dependent protection against uropathogenic *E. coli* ^9^ and display antibiofilm activity against methicillin-resistant *Staphylococcus aureus* (MRSA)^7^ and *Acinetobacter baumannii*.^35^ These findings demonstrate that HMOs possess broad antibiofilm and antiadhesive properties against both Gram-positive and Gram-negative species.^10^ For this reason, HMOs have been identified as an avenue of exploration for potential therapeutic use beyond their known nutritional benefits.

While HMOs are naturally consumed by infants, their diverse glycan structures also make them promising molecular probes for investigating glycan-mediated immune signaling in human cells. Importantly, HMO concentrations used in these *in vitro* studies are not intended to directly replicate intestinal exposure levels in infants, which are influenced by digestion, dilution, and transport processes. Instead, these controlled *in vitro* systems enable systematic evaluation of concentration-dependent cellular responses across a biologically relevant range, providing insight into how complex glycans influence inflammatory cytokine levels.

Consistent with this perspective, accumulating evidence indicates that HMOs modulate immune and inflammatory signaling. In a GBS infected pregnant mouse model, HMO treatment downregulated proinflammatory cytokines, including IL-1β, in the decidua, placenta, and fetus compared to untreated, GBS infected mice.^11^ The reduced bacterial burden, inflammation, and proinflammatory cytokine secretion in HMO treated mice was correlated with decreases in adverse pregnancy outcomes including preterm premature rupture of the membranes (PPROM), preterm birth, and maternal death compared to untreated, GBS infected mice.^11^ Treatment of LPS stimulated RAW264.7 macrophages with HMOs significantly reduced proinflammatory cytokines IL-1β and TNF-α.^12^ Similarly, HMOs enhanced immune recognition of *S. aureus* without triggering excessive inflammation in its absence.^13^ Collectively, these studies highlight the dual immunological capacity of HMOs to dampen pathological inflammation while enhancing pathogen specific immune responses. These properties hold promise for mitigating inflammation driven disorders, including those linked to preterm birth and chronic inflammatory disease. In the present work, HMOs are therefore examined not as nutritional components of infant feeding, but as bioactive glycans capable of modulating inflammatory signaling pathways in human macrophages.

Enzyme-linked immunosorbent assays (ELISAs) are highly valued for cytokine and immune biomarker quantification due to their robust specificity and established clinical validity. However, despite their reliability, ELISAs are often constrained by long assay times, high reagent consumption, and limited adaptability for real time or low volume applications, particularly in dynamic or 3D biological systems.^14^ Recent advancements in immunoassay technologies have introduced a variety of alternative platforms for immune and inflammation testing. Emerging methods such as photonic crystal biosensors,^15^ CRISPR-based diagnostic biosensors,^16^ and electrochemical impedance spectroscopy (EIS) sensors^17^ have demonstrated high sensitivity and specificity in detecting cytokines and other biomarkers. These platforms offer advantages like miniaturization and potential for multiplexing, making them suitable for point-of-care diagnostics. However, challenges remain with respect to complex fabrication processes, integration with biological models, and scalability for clinical applications.^18^ To address these limitations, the Cliffel laboratory developed a magnetic bead electrochemical sandwich assay (MBESA), combining the benefits of magnetic bead capture with electrochemical detection.^19^ The adoption of magnetic bead biosensors has the potential to improve the accessibility, precision, and throughput of immune and inflammation monitoring in both research and clinical settings.

Previous work has established MBESAs as a reliable and versatile platform for quantifying inflammatory cytokines in microscale and complex biological systems.^18^ Unlike traditional bead-based or electrode dependent electrochemical sensors, which often require surface modified electrodes, nanostructured interfaces, or intricate fabrication steps, MBESAs employ epoxy functionalized magnetic beads for target capture, coupled with a streptavidin-horseradish peroxidase (HRP)/TMB electrochemical readout performed off-bead.^18^ This modular design ensures consistent electron transfer kinetics, reduces variability introduced by on-electrode conjugation, and eliminates common interferences associated with self-assembled monolayers^20^ or prolonged blocking steps. Additionally, the assay operates effectively at low sample volumes, making it compatible with 3D or low yield biological models while remaining cost-effective.^21^

Leveraging these analytical advantages, we applied the MBESA platform to assess the immunomodulatory effects of HMOs, isolated from human breast milk, in THP-1 macrophages stimulated with LPS (Figure 1) to provide a sensitive and scalable approach for monitoring cytokine responses. While similar MBESA platforms have been previously reported for cytokine detection,^18^ the present study extends this analytical framework to quantify IL-1β and TNF-α in serum-containing macrophage cultures and applies the assay to investigate immunomodulatory effects of HMOs in human macrophages. The HMO concentrations evaluated were selected to systematically probe dose-dependent cytokine responses within a controlled *in vitro* macrophage system rather than to replicate physiological gastrointestinal exposure levels encountered during infant digestion. By examining HMO-mediated signaling across multiple concentrations, this work aims to characterize how structurally complex glycans influence macrophage inflammatory responses while simultaneously demonstrating the analytical sensitivity of the MBESA platform for resolving nuanced cytokine dynamics.

**Figure 1:**
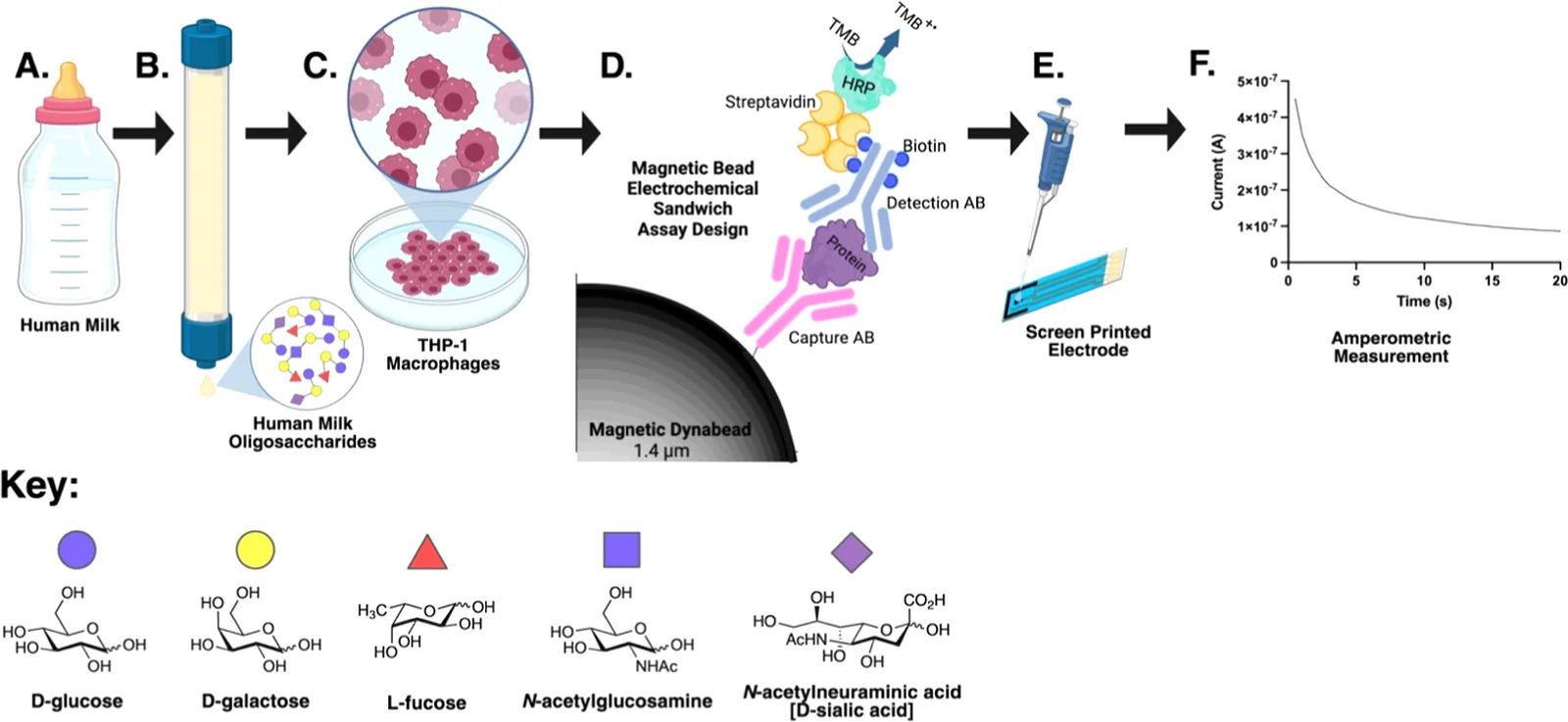
Workflow of the electrochemical platform to evaluate the immunomodulatory effects of heterogeneous HMOs isolated from human breast milk on cytokine responses in LPS-stimulated THP-1 macrophages. (A) Milk from human donors, (B) Size exclusion chromatography of human milk components, (C) THP-1 macrophage cells treated with HMOs and controls, (D) MBESA, (E) Screen-printed electrode, and (F) Amperometric (*i-t*) curve.

## Results and Discussion

### Performance of MBESA Linearity and Sensitivity for Key Inflammatory Cytokine Quantification

The MBESA operates through antibody–antigen recognition and enzyme-mediated signal amplification using magnetic bead capture and electrochemical detection. In this design, capture antibody-coated magnetic beads bind to the target protein in either a standard or sample solution, followed by the addition of a biotinylated detection antibody and streptavidin-HRP. Upon introducing TMB and hydrogen peroxide, HRP catalyzes a two step redox reaction that first produces a blue TMB radical cation intermediate and then a yellow diimine product. The oxidized TMB species are electrochemically reduced at the screen-printed electrode surface, generating a measurable current proportional to the amount of protein in the sample.^18^ Since the MBESA relies on the universal principles of antibody specificity and HRP/TMB electrochemistry, the platform can be easily adapted to quantify various inflammatory cytokines, including IL-1β and TNF-α, by substituting the capture and detection antibodies specific to a new target. This modular design allows for flexible and reproducible quantification of a wide range of proteins using the same magnetic bead and electrochemical framework.

The MBESA was calibrated for IL-1β and TNF-α in RPMI media supplemented with 10% FBS and 1% AA solution to evaluate performance in a complex biological environment (Figure 2). For IL-1β, assays demonstrated a clear linear relationship between electrochemical charge and protein concentration over the 25–800 pg/mL range, with triplicate measurements confirming reproducibility. Multiple calibration curves were established to validate consistency and optimal electrochemical performance across measurments. IL-1β calibration curve A produced an *R* ^2^ of 0.9940, a slope coefficient of variation (CV) of 4%, and an intercept CV of 48%, while the limit of detection (LOD) and limit of quantification (LOQ) were 2 pg/mL and 6 pg/mL, respectively (Figure 2A). Similarly, IL-1β calibration curve C produced an *R* ^2^ of 0.9958, a slope CV of 3%, and an intercept CV of 26%, while the LOD and LOQ were 2 pg/mL and 7 pg/mL, respectively (Figure 2C). Importantly, these independent calibration curves yielded highly consistent analytical performance, confirming the robustness and reproducibility of the MBESA platform across measurements. Notably, the improved intercept coefficient of variation observed in calibration curve C further demonstrates enhanced baseline stability and analytical precision following optimization.

**Figure 2:**
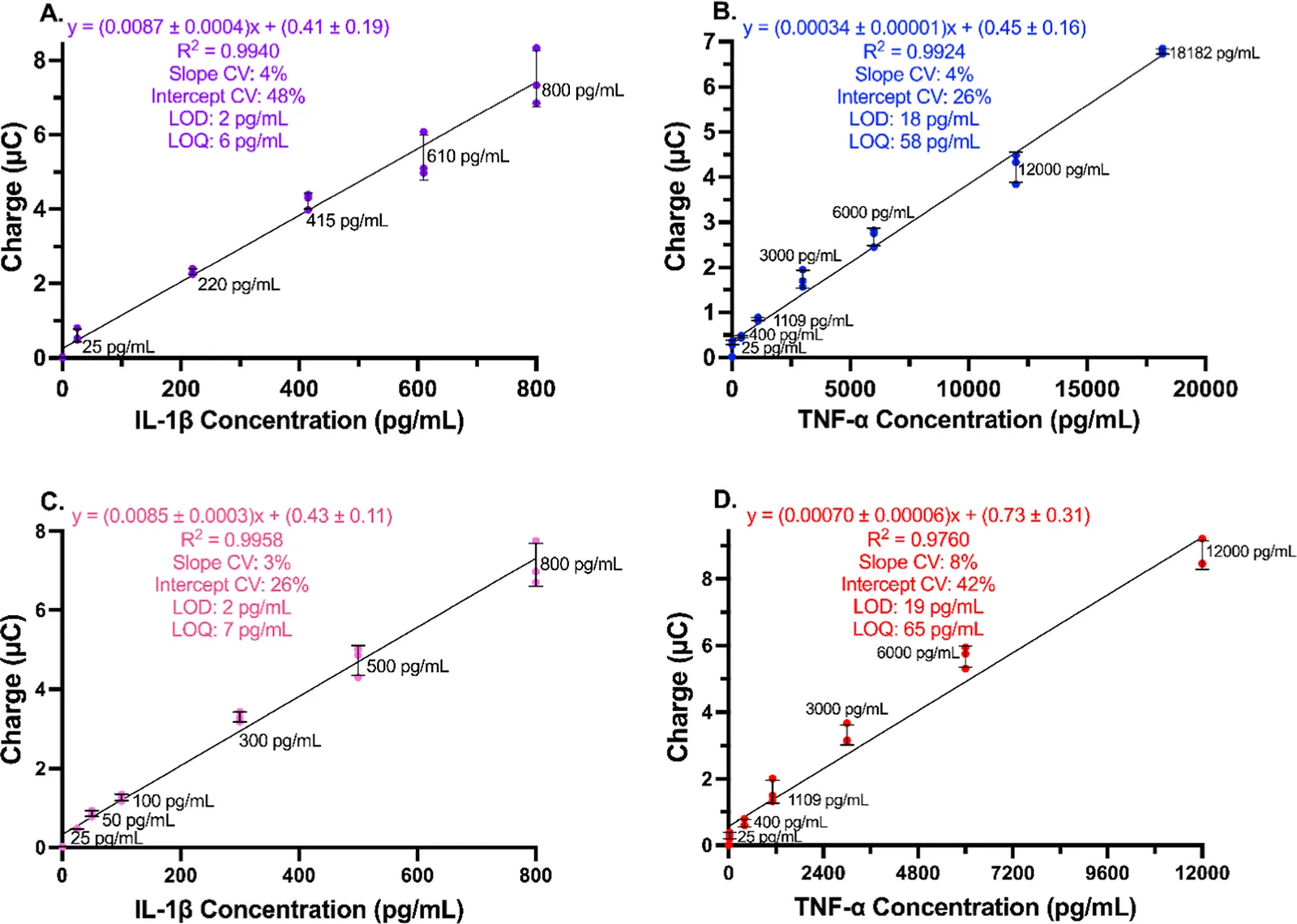
Electrochemical charge vs cytokine concentration triplicate calibrations in macrophage RPMI media with 10% fetal bovine serum (FBS) and 1% antibiotic/antimycotic (AA) for IL-1β (A and C) and TNF-α (B and D). Calibrations A and B were performed with 5 μg target capture antibody per 1 mg bead, 2 μL of 250 ng/mL detection antibody and 8 min of 3,3′,5,5′-tetramethylbenzidine (TMB) incubation. Optimized calibrations C and D were performed with 3 μg target capture antibody per 1 mg bead, 2.5 μL of 1000 ng/mL detection antibody and 2.5 min of TMB incubation. *R* ^2^, slope CV, and intercept CV were within accepted values for all cytokine calibrations.

The TNF-α calibration covered a broader range, from 25 pg/mL up to 18200 pg/mL, yielding an *R* ^2^ of 0.9924, slope CV of 4%, intercept CV of 26%, LOD of 18 pg/mL, and LOQ of 58 pg/mL (Figure 2B). An additional calibration curve from 25 pg/mL up to 12000 pg/mL, yielded an *R* ^2^ of 0.9760, slope CV of 8%, intercept CV of 42%, LOD of 19 pg/mL, and LOQ of 65 pg/mL (Figure 2D). Importantly, these calibration curves demonstrate precise quantification across a wide dynamic range, with minor variation in analytical parameters, supporting the robustness of the MBESA platform. In both cases of TNF-α and IL-1β, electrochemical charge increased proportionally with cytokine concentration, confirming the linearity and sensitivity of the MBESA in serum-containing media. These validated calibration curves were then applied to quantify precise TNF-α and IL-1β levels present in HMO and LPS treated THP-1 macrophage samples, enabling extremely sensitive assessment of cytokine responses across multiple treatment conditions.

### HMO Treatment Differentially Modulates Cytokine Secretion in THP-1 Macrophages

Biological application of the MBESA was compared and validated to traditional ELISAs, with no significant difference found between the two techniques.^18^ To assess the immunomodulatory effects of HMOs using the MBESA, IL-1β and TNF-α secretion were quantified from differentiated THP-1 macrophages following treatment with HMOs (1, 5, and 15 mg/mL) in the absence or presence of LPS (1 μg/mL) (Figure 3).

**Figure 3:**
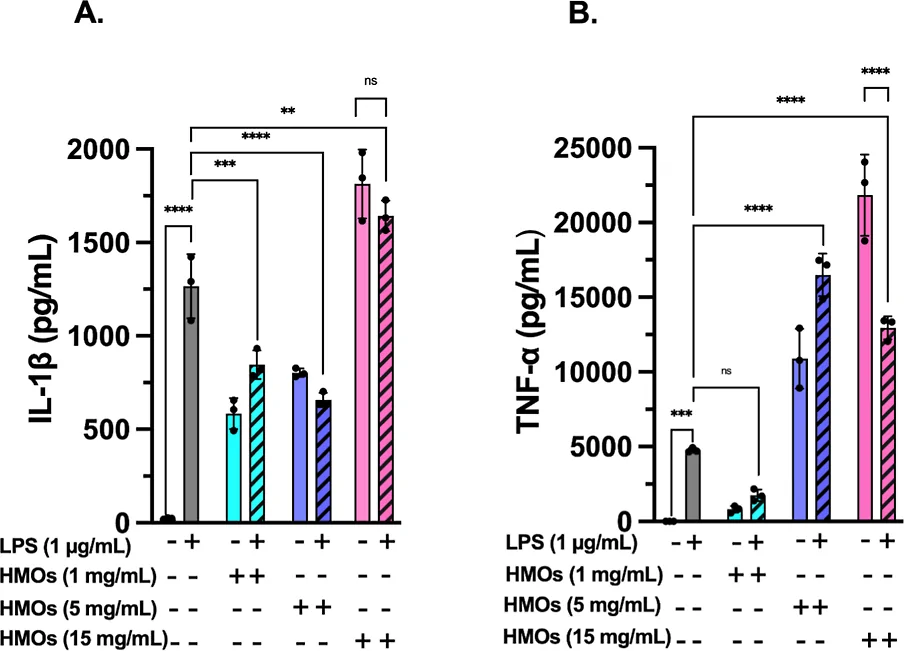
Human milk oligosaccharides suppress LPS-induced IL-1β secretion (A) and increase TNF-α secretion (B) in PMA-differentiated THP-1 macrophages. Cells were treated for 24 h with RPMI, LPS (1 μg/mL), HMOs (1 mg/mL), HMOs (5 mg/mL), HMOs (15 mg/mL), LPS (1 μg/mL) +1 mg/mL HMOs, LPS (1 μg/mL) +5 mg/mL HMOs, or LPS (1 μg/mL) +15 mg/mL HMOs. Supernatant cytokines were quantified by MBESA. Data are mean ± SEM (*n* ≥ 3). ****p < 0.0001; ***p < 0.001; **p < 0.01; *p < 0.05 vs indicated groups; ns, not significant. Analyses were performed with two-way ANOVA and Tukey’s multiple comparisons. Bar colors indicate different treatment conditions, while diagonal lines denote samples cotreated with HMOs and LPS.

### IL-1β Secretion Exhibits a Biphasic Response to HMO Treatment

Unstimulated control cells exhibited minimal IL-1β secretion (23.7 ± 4.1 pg/mL), whereas LPS stimulation alone markedly increased IL-1β to 1270 ± 170 pg/mL (*p* < 0.0001). Treatment with HMOs alone (in the absence of LPS) induced IL-1β secretion in a dose dependent manner. At the lowest concentration tested, 1 mg/mL HMOs increased IL-1β to 585 ± 83 pg/mL, followed by 5 mg/mL HMOs which raised levels to 803 ± 23 pg/mL, and 15 mg/mL HMOs which produced the highest response of 1810 ± 180 pg/mL, surpassing LPS alone by 43%.

When coadministered with LPS, the effect of HMOs was strikingly concentration dependent. At 1 mg/mL, HMOs in combination with LPS resulted in IL-1β levels of 846 ± 77 pg/mL, representing a partial reduction relative to LPS alone. At 5 mg/mL, HMOs significantly suppressed LPS induced IL-1β secretion to 660 ± 40 pg/mL, representing a 48% reduction relative to LPS alone (*p* < 0.0001). In contrast, the higher dose (15 mg/mL) enhanced IL-1β levels, yielding 1640 ± 80 pg/mL, which is 30% greater than LPS alone (*p* < 0.01) and only marginally lower than 15 mg/mL HMOs without LPS. Statistical analysis using two-way ANOVA demonstrated that both HMO treatment alone (p < 0.001) and cotreatment of HMOs and LPS (*p* < 0.001) significantly influenced IL-1β secretion. However, Tukey’s multiple comparisons testing indicated that IL-1β levels did not differ significantly between HMO only and cotreated groups at 5 mg/mL or 15 mg/mL HMO concentrations. Additionally, IL-1β secretion was not significantly different between the 1 and 5 mg/mL HMO concentrations within either treatment condition.

Thus, IL-1β secretion shows a biphasic dose response to HMO treatment in the context of LPS stimulation. Lower HMO concentrations reduced LPS-induced IL-1β production, with 5 mg/mL HMOs suppressing secretion by nearly 50%. In contrast, the highest concentration tested (15 mg/mL) shifted the response toward enhanced IL-1β production, suggesting a potential proinflammatory effect at elevated HMO concentrations. This suppression may reflect modulation of inflammasome-associated pathways, such as NOD-like receptor family pyrin domain containing 3 (NLRP3) inflammasome activation, caspase-1 processing, or upstream toll-like receptor 4 (TLR4)/nuclear factor-κB (NF-κB) priming, as suggested by prior studies examining HMO-mediated immune signaling.^26^ Consistent with this possibility, reports have shown that sialylated and fucosylated HMOs can disrupt TLR4/NF-κB/NLRP3 signaling cascades.^27,28^ In fact, previous work indicated that sialylated HMOs were critical for preventing NF-κB activation in response to uropathogenic *E. coli*.^9^ The reversal at higher concentrations may indicate a threshold-dependent shift in immune signaling, potentially promoting secondary danger pathways that favor inflammasome assembly.^29–31^ Since the scope of these studies included quantifying secreted cytokine responses using the MBESA platform, future investigations will include direct validation of these pathways with targeted molecular analyses.

Additionally, while the HMO preparation underwent purification prior to use and microscopic observation of macrophages following all treatments did not reveal signs of cell death, the present study did not directly assess cell viability, osmotic stress, or pyroptosis-associated cytokine release. Future investigations incorporating this analysis will be valuable for distinguishing direct immunomodulatory signaling from cellular stress responses. Overall, these findings highlight that HMO-mediated immune modulation of IL-1β is highly concentration dependent and emphasize the importance of dose selection in potential therapeutic applications.

### TNF-α Secretion is Enhanced by HMO Treatment

Baseline TNF-α was undetectable in unstimulated control cells, whereas LPS stimulation alone induced robust secretion (4760 ± 150 pg/mL, *p* < 0.001). Treatment with HMOs alone (in the absence of LPS) dramatically increased TNF-α in a concentration dependent manner. At 1 mg/mL, HMOs induced TNF-α secretion to 817 ± 224 pg/mL, followed by 5 mg/mL HMOs which raised levels to 11000 ± 2000 pg/mL (128% increase over LPS alone), while 15 mg/mL HMOs produced the highest response observed in the entire study (21900 ± 800 pg/mL), representing a 4.6 fold increase versus LPS alone. It is worth highlighting that the lowest dose, 1 mg/mL HMOs, produced TNF-α levels below those induced by LPS alone, but this reduction was not statistically significant.

When coadministered with LPS, HMOs continued to drive TNF-α secretion, although the response peaked at the intermediate concentration of 5 mg/mL. At 1 mg/mL, HMOs in combination with LPS induced TNF-α secretion to 1753 ± 392 pg/mL, exceeding unstimulated HMO treatment alone, but presenting a reduction compared to LPS alone. However, neither comparison was statistically significant. The intermediate dose (5 mg/mL) acted mutually with LPS to elevate TNF-α to 16500 ± 1400 pg/mL (250% increase vs LPS alone, *p* < 0.0001). The highest dose (15 mg/mL) still markedly increased TNF-α compared to LPS alone (12900 ± 800 pg/mL; 170% increase, *p* < 0.0001), but this represented a notable 41% reduction relative to 15 mg/mL HMOs without LPS (*p* < 0.0001). Statistical analysis using two-way ANOVA demonstrated that both HMO treatment alone (*p* < 0.001) and cotreatment of HMOs and LPS (*p* < 0.001) significantly influenced TNF-α secretion. Tukey’s multiple comparisons testing between HMO only groups and cotreated groups further confirmed significant differences between all treatment conditions, except for the 1 mg/mL HMO treatment and 1 mg/mL HMO cotreatment with LPS.

In contrast to the biphasic response observed for IL-1β, HMO treatment alone produced a concentration dependent increase in TNF-α secretion, as higher concentrations produced amplified responses. However, under LPS stimulated conditions, TNF-α secretion peaked at 5 mg/mL HMOs and declined at 15 mg/mL, indicating a nonlinear response between HMO concentration and inflammatory stimulation. This elevation suggests that HMOs may influence signaling pathways associated with transcriptional cytokine activation, such as NF-κB-driven responses reported in previous studies of glycan-mediated immune modulation.^32^ However, since the present study quantified end point cytokine secretion rather than intracellular signaling events, these associations are plausible hypotheses rather than direct mechanistic conclusions. Furthermore, the partial reduction observed when high dose HMOs were combined with LPS, while still remaining far above LPS alone, suggests context specific negative feedback, receptor competition, or signaling saturation when both stimuli are present simultaneously.^33^ These findings align with reports that specific fucosylated and neutral HMOs engage TLR4 or C-type lectin receptors to prime macrophages and boost TNF-α as part of physiological immune vigilance rather than pathological inflammation.^11,13^ Collectively, the data suggests HMOs alone are potent and consistent enhancers of TNF-α production, capable of dramatically amplifying secretion both alone and in cooperation with microbial signals.

### HMOs Induce Cytokine Specific and Dose Dependent Responses

This study demonstrates that HMOs exert cytokine specific and dose dependent effects on macrophage inflammatory signaling. Notably, previous studies in which HMO-mediated decreases in IL-1β and TNF-α were observed utilized immortalized mouse macrophages,^12^ whereas the present study was performed in human cells, highlighting a potential species or model specific difference. The observed divergence in IL-1β and TNF-α responses indicates that HMOs do not uniformly suppress or activate inflammation but instead function as dynamic immune modulators capable of reshaping cytokine profiles depending on concentration and inflammatory context.

Rather than acting as traditional anti-inflammatory agents, HMOs appear to recalibrate inflammatory balance. At the lowest concentration (1 mg/mL), HMOs produced modest reductions in IL-1β and TNF-α, while the intermediate dose (5 mg/mL) continued to suppress IL-1β but amplified TNF-α. In contrast, 15 mg/mL HMOs in the absence of LPS, consistently enhanced the production of both cytokines, suggesting that these glycans function as dose dependent immunomodulators rather than unidirectional suppressants. Notably, this dual behavior may reflect a transition from regulatory immune modulation to heightened macrophage activation under high-concentration HMO exposure, which falls within physiologically relevant ranges (5–25 mg/mL)^5^ where HMOs would be present prior to intestinal absorption and metabolism. Importantly, the divergence between IL-1β and TNF-α responses highlights that cytokine regulation by HMOs is not generalized but pathway-specific. These insights suggest that individual oligosaccharide composition, molecular structure, and concentration will be critical determinants of immune outcome.

In this work, HMOs exert distinct, context dependent effects on macrophage inflammatory signaling, shaping both baseline and LPS-induced cytokine release in differentiated THP-1 cells. When macrophages were challenged with LPS, 1 and 5 mg/mL HMOs significantly blunted IL-1β secretion, further supporting their capacity to recalibrate inflammasome related inflammatory output during pathogen associated stimulation. The increased inflammation observed in the presence of 15 mg/mL is likely attributed to the concentration dependent effects of HMOs, with lower concentrations shown to induce less inflammation. However, the downregulation in TNF-α and IL-1β production in the 15 mg/mL and LPS infected cells may still be a promising indication that HMOs can augment immune responses against infections.

### Characterization and Application of Heterogeneous HMO Mixture

For this investigation, a heterogeneous mixture of HMOs was employed rather than isolated oligosaccharides. This was informed by prior work demonstrating that HMO mixtures exhibit enhanced biological activity compared to individual components, particularly in the context of antimicrobial function.^6^ Notably, studies from the Townsend laboratory have shown that no single HMO replicates the antimicrobial efficacy of the native heterogeneous mixture.^6^ While these antimicrobial mechanisms involve pathogen binding interference and disruption of microbial adhesion or growth,^27^ such pathways are mechanistically distinct from immunomodulation. Therefore, although antimicrobial activity has been attributed to specific HMOs, these effects are mechanistically distinct from direct modulation of host immune signaling pathways.

Nevertheless, individual HMOs are known to exert distinct and sometimes opposing effects on immune responses, raising the possibility that the cytokine modulation observed here reflects the combined influence of specific bioactive components within the mixture. Prior studies report that 2′-fucosyllactose (2′-FL), lacto-*N*-neotetraose (LNnT), and 6′-sialyllactose (6′-SL) can induce pro-inflammatory cytokines such as TNF-α and IL-1β under infectious conditions,^13^ whereas other HMOs, including 3′-sialyllactose (3′-SL) and 2′-FL in alternative cellular contexts, have been associated with anti-inflammatory signaling, including induction of interleukin-10 (IL-10).^28^ These findings suggest that 2′-FL and related HMOs may not be intrinsically pro- or anti-inflammatory but instead exert context and dose dependent effects on immune signaling. For this reason and to increase reproducibility, a characterization of the heterogeneous HMO mixture used in this study is included in the Supporting Information.

Previous characterization of the Townsend laboratory heterogeneous HMO mixtures has detected the presence of 3-fucosyllactose (3-FL), 2′-fucosyllactose (2′-FL), difucosyllactose (DFL), lacto-*N*-fucopentasose I (LNFP I), lacto-*N*-fucopentasose II (LNFP II), lacto-*N*-fucopentasose III (LNFP III), lacto-*N*-triose II (LNT II), lacto-*N*-neohexaose (LNnH), *para*-lacto-*N*-neohexaose (*para*-LNnH), lacto-*N*-tetraose (LNT), and lacto-*N*-neotetraose (LNnT).^22,23^ In this study, characterization of HMOs from multiple donors identified all the aforementioned HMOs except LNnH and *para*-LNnH (Figures S1 and S2). In naturally occurring HMO mixtures, 35–50% of HMO structures are fucosylated and 10–15% are sialylated.^34^ Consistent with these trends, 2′-FL is known to be the most abundant HMO overall, while LNnT is one of the most abundant neutral HMOs, and 3′-SL and 6′-SL are among the most abundant sialylated HMOs.^35^ Given the intentional heterogeneity of the HMO cocktail used in this study, the mixture is expected to mimic these naturally occurring compositional ratios. This structural diversity provides a more physiologically relevant model for studying glycan-mediated immunomodulation and further contextualizes the cytokine responses observed here.

Biological application of this heterogeneous mixture produced a nonlinear biphasic IL-1β response and a sustained enhancement of TNF-α secretion across all HMO concentrations. These patterns suggest that the net immunomodulatory outcome likely reflects integrated activity of multiple bioactive HMO structures, rather than the action of a single dominant oligosaccharide. Future work could therefore focus on isolating and evaluating individual HMOs to directly compare their immunomodulatory effects with those of the heterogeneous mixture used in this study. Systematic assessment of purified HMOs across a range of concentrations will enable determination of whether the observed dual immunomodulatory behavior arises from combined interactions within the mixture or from dose dependent effects of specific HMOs, such as 2′-FL. Such studies will be critical for unveiling HMO specific immune signaling mechanisms and for guiding rational design of glycan based immunomodulatory strategies. Achieving these goals will require analytical platforms capable of resolving subtle, concentration dependent cytokine responses with high sensitivity and reproducibility.

### Utility of MBESA Application

To further contextualize these findings, all cytokine measurements were collected at a 24 h time point, which represents a well-established window for capturing both transcriptionally regulated cytokines such as TNF-α and inflammasome associated IL-1β secretion. Importantly, the sensitivity of the MBESA platform enabled detection of subtle, yet biologically meaningful cytokine changes at this early stage of immune activation. This capability allows for resolution of concentration dependent immunomodulatory effects that may not be readily apparent using less sensitive analytical techniques. While additional time points and post-treatment designs may further resolve temporal dynamics, the present study leverages this standardized end point to enable direct comparison across treatment conditions while demonstrating the analytical strength of the platform.

The MBESA was pivotal in capturing these nuanced cytokine shifts with greater precision and sensitivity than conventional ELISAs. Although bead-based electrochemical platforms have been explored, their application to characterize glycan-mediated immune modulation in macrophage systems remains limited. This work therefore extends the use of electrochemical assays to investigate inflammatory signaling and contributes to the growing body of literature exploring electrochemical platforms, as an alternative to the ELISA gold-standard method, for sensitive cytokine quantification in complex biological environments. Additionally, the multiplexing capability and low volume requirements of the MBESA enabled profiling of IL-1β and TNF-α secretion, demonstrating its potential as a high-resolution tool for studying macrophage dynamics and screening immunomodulatory compounds.

Beyond these practical advantages, MBESAs leverage electrochemical detection, which provides fundamental benefits for inflammatory analysis such as rapid electron-transfer based readouts, low detection limits, and excellent signal-to-noise ratio. These features make electrochemical assays particularly well suited for tracking subtle or transient cytokine changes that may be missed by optical methods, offering a robust platform for investigating macrophage signaling and the effects of bioactive compounds such as HMOs. Lastly, while the present study validated the MBESA platform in RPMI media supplemented with 10% FBS, the electrochemical design is well suited for application in highly complex biological matrices. This feature will support future translation of the platform toward clinical samples, including human plasma,^19^ cervical fluid, or amniotic fluid, where rapid and sensitive cytokine monitoring could provide high valuable insight into adverse pregnancy outcomes. By integrating electrochemical detection with biologically relevant immune readouts, the MBESA functions as a chemistry driven diagnostic platform that bridges analytical chemistry and host–pathogen biology.

## Conclusions

While these findings reveal clear modulatory effects of HMOs, this study is limited by using a single *in vitro* macrophage model. Since immortalized cell lines do not fully recapitulate the complexity of primary human immune responses, the cytokine patterns observed here should not be interpreted as universally accepted immune behavior. Future work integrating primary cells, coculture systems, and mechanistic pathway analyses will be essential to define receptor specific engagement, dose thresholds, and structure function relationships governing HMO driven immune signaling. Such studies will be essential for translating *in vitro* mechanistic insights into therapeutic strategies for controlling inflammation.

Ultimately, this work underscores HMOs as potent, concentration dependent regulators of macrophage inflammatory responses and further demonstrates the utility of the MBESA as a sensitive electrochemical alternative to traditional immunoassays for studying subtle dynamics in complex biological systems. By integrating sensitive cytokine monitoring with functional immune assays, these findings provide a framework for advancing inflammation research and deepen our understanding of how dietary glycans can shape immune signaling. Furthermore, this study advances HMO research by demonstrating their dual immunomodulatory capacity and positioning them as promising candidates for future mechanistic and therapeutic investigations.

## Experimental Section

### Human Milk Oligosaccharide Isolation

Human milk from lactating patients between 3 days and three months postnatal was provided by Dr. Jorn-Hendrik Weitkamp from the Vanderbilt Department of Pediatrics under a collection protocol approved by the Vanderbilt University Institutional Review Board (IRB #100897). Milk samples stored at −20 °C were thawed and then centrifuged for 45 min. The top lipid layer was then removed. The remaining sample was diluted with 2:1 v/v 70% ethanol, chilled for 5 min on dry ice and then centrifuged for 45 min to remove proteins. The supernatant was concentrated *in vacuo*, dissolved in phosphate buffer (pH 6.5, 0.2 M), then β-galactosidase from *Kluveromyces lactis* was added and the sample was heated to 40 °C in an oil bath. The reaction was stirred until lactose hydrolysis was complete, as visualized by thin layer chromatography, and then the solution was diluted with 1:1 v/v 70% ethanol. The sample was chilled for 5 min on dry ice, then centrifuged for 30 min. The resulting supernatant was concentrated *in vacuo*. The oligosaccharides were isolated from the remaining salts, glucose, and galactose via P-2 Gel (H_2_O eluent) size exclusion chromatography and then dried by lyophilization.^5,7^ HMOs from 9 healthy donors were rehydrated in sterile distilled water and pooled to create a stock of 102.6 mg/mL concentration of HMOs. This HMO cocktail was aliquoted into 1 mL tubes and frozen at −20 °C until use in biological assays. The HMO cocktail has previously been extensively characterized via matrix-assisted laser desorption/ionization Fourier transform ion cyclotron resonance mass spectrometry (MALDI-FT-ICR MS) and tandem mass spectrometry MS/MS.^5,7,11,22–25^ Individual donor samples have been analyzed via ^1^H and ^13^C NMR.^25^ Two donors used in the HMO cocktail for this study were analyzed by MALDI trapped ion mobility spectrometry time-of-flight MS (MALDI-tims-TOF MS and MS/MS (Figures S1 and S2).

### THP-1 Macrophage Preparation, THP-1 Macrophage Preparation

The human monocytic leukemia cell line THP-1 (ATCC Cat. #TIB202) was cultured in Roswell Park Memorial Institute 1640 medium supplemented with 2 mM l-glutamine (RPMI 1640; Gibco Cat. #11875093), 10% charcoal-stripped fetal bovine serum (Gibco Cat. #12676029), and 1% antibiotic/antimycotic solution (Gibco Cat. #15240062). To induce differentiation into macrophage-like cells, nonadherent THP-1 monocytes were treated with 5 ng/mL phorbol 12-myristate 13-acetate (PMA; Sigma-Aldrich Cat. #P8139) on the day prior to plating for subsequent LPS and HMO stimulation experiments.

### HMO and LPS Treatment

THP-1 cells were differentiated with PMA and seeded at a density of 1 × 10^6^ cells per well, then allowed to adhere overnight. Cells were subsequently exposed to either an RPMI media control, 1 μg/mL ultrapure *lipopolysaccharide* derived from *E. coli* K12 (InvivoGen, Cat. #tlrl-3pelps), 1 mg/mL, 5 mg/mL, or 15 mg/mL of isolated HMOs, both 1 μg/mL LPS and 1 mg/mL HMOs, 1 μg/mL LPS and 5 mg/mL HMOs, or 1 μg/mL LPS and 15 mg/mL HMOs for 24 h. Following treatment, culture supernatants were collected and stored for downstream analysis.

### Magnetic Bead Electrochemical Sandwich Assay

All MBESAs were first validated in phosphate buffered saline (PBS) before being performed in biological culture supernatants and assay development was based upon our previously established methods.^18,19^ To prepare M-270 Epoxy Dynabeads, capture antibodies (5 μg target capture antibody per 1 mg beads) were coupled to Dynabeads M-270 Epoxy beads using the Dynabeads Antibody Coupling Kit (Invitrogen Cat. #14311D) following manufacturer instructions. Beads and antibodies were incubated overnight at 37 °C with gentle mixing. After incubation, beads were washed according to the kit protocol and resuspended to generate a stock solution. Aliquots of the antibody-coated beads were prepared for assays and stored at 4 °C until use.

Target protein standards or sample supernatants (20 μL standard/sample volume) were incubated with 2 μL of 250 ng/mL detection antibody and antibody-coated magnetic beads for 30 min at room temperature with gentle mixing. Beads were then magnetically separated and washed twice sequentially with phosphate buffered saline +0.1% Tween 20 (PBST) and last PBS to remove unbound material, ensuring minimal bead loss throughout the rinsing steps. Poly-HRP streptavidin (15 μL of 4 μg/mL; Thermo Fisher Cat. #N200) was added to the beads, gently mixed for 5 min, and washed twice with PBST and once with PBS. Beads were then incubated with 100 μL of TMB substrate (Millipore Sigma Cat. #T0440) for 8 min, and then the TMB solution was immediately used for electrochemical measurement. To optimize electrochemical signal output, assays were also prepared with 3 μg target capture antibody per 1 mg beads, 2.5 μL of 1000 ng/mL detection antibody, and incubated with 100 μL of TMB substrate for 2.5 min.

Electrochemical analyses were performed using a CHI 1440 4-Channel Potentiostat (CH Instruments, Inc., Austin, TX, USA). Amperometric measurements were carried out with the following settings: potential of −0.2 V, sample interval of 0.5 s, total sampling time of 20 s, quiet time of 0 s, and a sensitivity of 1 × 10^–5^ A/V. Carbon screen-printed electrodes (Pine Research Instrumentation, Inc., Durham, NC, USA) were used, consisting of a 2 mm diameter carbon working electrode, a carbon counter electrode, and an Ag/AgCl reference electrode. Sensor performance was assessed by recording amperometric current versus time (*i*-*t*) curves (Figure S3). The recorded *i*-*t* curves were then integrated to calculate the total charge, which was used as the quantitative output of the assay.

### Interleukin-1β Electrochemical Assay Preparation

Lyophilized IL-1β (Invitrogen Cat. #CHC1213) was reconstituted in RPMI 1640 supplemented with 10% charcoal-stripped fetal bovine serum and 1% antibiotic/antimycotic solution to create a 10 ng/mL stock. The stock was then diluted to generate protein standards of 25 pg/mL, 50 pg/mL, 100 pg/mL, 220 pg/mL, 300 pg/mL, 415 pg/mL, 500 pg/mL, 610 pg/mL, and 800 pg/mL, encompassing the physiological IL-1β range expected from THP-1 macrophages.

### Tumor Necrosis Factor-α Electrochemical Assay Preparation

Lyophilized TNF-α (Invitrogen Cat. #CHC1715) was reconstituted in RPMI 1640 supplemented with 10% charcoal-stripped fetal bovine serum and 1% antibiotic/antimycotic solution to create an 18 ng/mL stock. The stock was then diluted to generate protein standards of 25 pg/mL, 400 pg/mL, 1100 pg/mL, 3000 pg/mL, 6000 pg/mL, 12000 pg/mL, and 18200 pg/mL, encompassing the physiological TNF-α range expected from THP-1 macrophages.

### Statistical Analysis

Data were analyzed using GraphPad Prism 10.0. A two-way ANOVA and Tukey’s multiple comparisons test were applied, with *p*-values <0.05 considered statistically significant (α = 0.05, *n* ≥ 3). Individual data points from independent biological experiments are presented alongside mean values.

## Supplementary Material



## Data Availability

All data generated or analyzed in this study are included within this article and are accessible to any researcher for the purposes of replication or further analysis.

## Notes

Table detailing structural description of molecular ions observed in individual donor samples of breast milk, MALDI-tims-TOF MS/MS spectra for HMO samples from two donors used in the HMO cocktail for this work, example of amperometric current versus time (*i*-*t*) curve and integrated curve used to generate data presented in Figure 2 (PDF)

## Abbreviations

IL-10: interleukin-10
3-FL: 3-fucosyllactose
2′-FL: 2′-fucosyllactose
DFL: difucosyllactose
LNFP I: lacto-N-fucopentasose I
LNFP II: lacto-N-fucopentasose II
LNFPIII: lacto-N-fucopentasose III
LNT II: lacto-N-triose II
LNnH: lacto-N-neohexaose
para-LNnH: para-lacto-N-neohexaose
LNT: lacto-N-tetraose
LNnT: lacto-N-neotetraose
6′-SL: 6′-sialyllactose
3′-SL: 3′-sialyllactose
PBS: phosphate buffered saline
IL-1β: interleukin-1β
MBESA: magnetic bead electrochemical sandwich assay
ELISA: enzyme-linked immunosorbent assay
POC: point-of-care
HRP: horseradish-peroxidase
TMB: 3,3′,5,5′-tetramethylbenziidine
HMOs: human milk oligosaccharides
TNF-α: tumor necrosis factor-α
LPS: lipopolysaccharide
*E. coli*: *Escherichia coli*
GBS: Group B *Streptococcus*
MRSA: methicillin-resistant *Staphylococcus aureus*
EIS: electrochemical impedance spectroscopy
MALDI-FT-ICR MS: matrix-assisted laser desorption/ionization Fourier transform ion cyclotron resonance mass spectrometry
MS/MS: tandem mass spectrometry
MALDI-tims-TOF MS: matrix-assisted laser desorption/ionization trapped ion mobility spectrometry time-of-flight mass spectrometry
RPMI: Roswell Park Memorial Institute
PMA: phorbol 12-myristate 13-acetate
PBST: phosphate buffered saline +0.1% Tween 20
FBS: fetal bovine serum
CV: coefficient of variation
LOD: limit of detection
LOQ: limit of quantification
NLRP3: Nod-like Receptor Pyrin Domain-containing 3
TLR4: Toll-Like Receptor 4
NF-κB: nuclear factor, kappa-light-chain-enhancer of activated B cells.
