# Supplementary material for: Electrochemical Cytokine Profiling Reveals Dual Immunomodulatory Effects of Human Milk Oligosaccharides in THP‑1 Macrophages
Source: ACS Infect Dis. 2026 Sep 30;12(10):3398–407. doi: 10.1021/acsinfecdis.6c00388 (PMC13660392; doi:10.1021/acsinfecdis.6c00388)
Supplement: Supplementary file 1 [file id6c00388_si_001.pdf]

Supporting Information

Electrochemical cytokine profiling reveals dual immunomodulatory effects of human milk oligosaccharides in THP-1 macrophages

*Victoria A. Federico[a], Hannah A. Richards[a], Julie A. Talbert[a], Jocelyn R. Leal[b], Jennifer A. Gaddy[c], Steven D. Townsend[a], David E. Cliffel[a]**

[a]Department of Chemistry, Vanderbilt University, Tennessee 37235-1822, United States

[b]Department of Chemistry, Haverford College, 370 Lancaster Ave, Haverford, PA 19041, United States

[c]Department of Medicine, Vanderbilt University Medical Center, Tennessee 37232-2625, United States

*Corresponding Author: David E. Cliffel – d.cliffel@vanderbilt.edu

Victoria A. Federico – Department of Chemistry, Vanderbilt University, Tennessee 37235-1822, United States; https://orchid.org/0009-0003-6330-1310

Hannah A. Richards – Department of Chemistry, Vanderbilt University, Tennessee 37235-1822, United States; https://orcid.org/0000-0002-5472-398X

Julie A. Talbert – Department of Chemistry, Vanderbilt University, Tennessee 37235-1822, United States;

Jocelyn R. Leal – Department of Chemistry, Haverford College, 370 Lancaster Ave, Haverford, PA 19041, United States;

Jennifer A. Gaddy- Department of Medicine, Vanderbilt University Medical Center, Tennessee 37232-2625, United States; https://orcid.org/0000-0002-2192-4224

Steven D. Townsend – Department of Chemistry, Vanderbilt University, Tennessee 37235-1822, United States; https://orcid.org/0000-0001-5362-7235

David E. Cliffel - Department of Chemistry, Vanderbilt University, Tennessee 37235-1822, United States; https://orcid.org/0000-0001-8756-106X; Email: d.cliffel@vanderbilt.edu

SUPPPORTING INFORMATION

HMO Characterization. As previously reported[30], HMO exact mass was calculated by using a molecular formula with the known masses of the most abundant isotopes of the elements in a particular structure (**Table S1.**). Values for [M + Na$^+$] were calculated with the mass of the sodium adduct expected after ionization. In this study, HMOs from multiple donors added to the heterogenous mixture were analyzed by MALDI-tims-TOF MS/MS and common HMO structures were identified (**Figures S1** and **S2**).

Table S1. Structural description of molecular ions observed in individual donor samples of breast milk.

**HMO Structure**	**Exact Mass**	**[M + Na$^+$]**
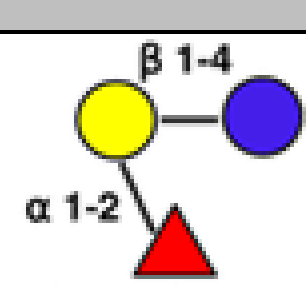 2'-fucosyllactose (2'-FL)	488.17	511.16

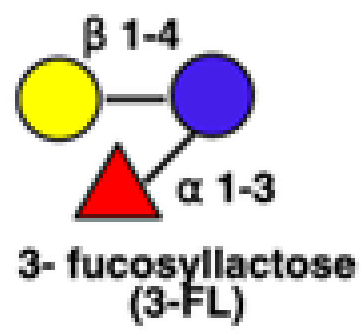	488.17	511.16
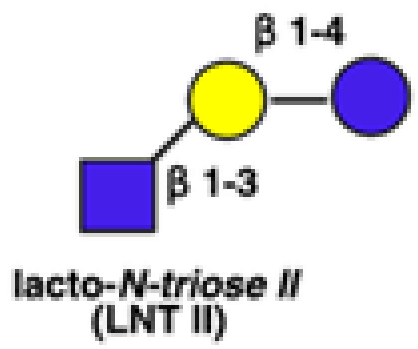	545.20	568.19
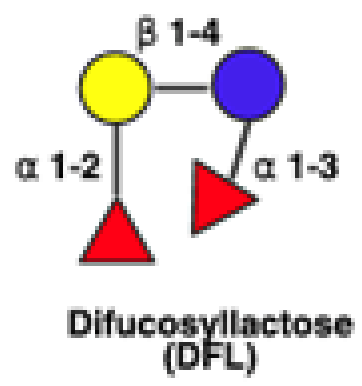	634.23	657.22
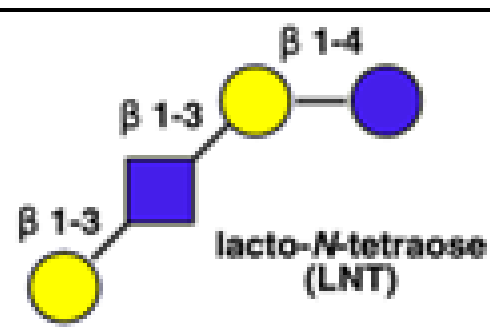	707.25	730.24
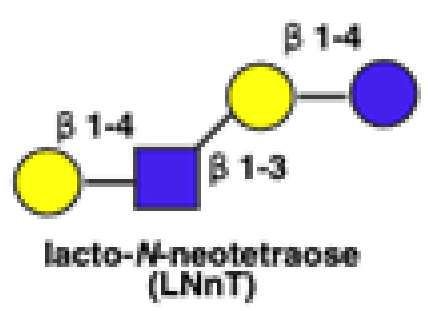	707.25	730.24
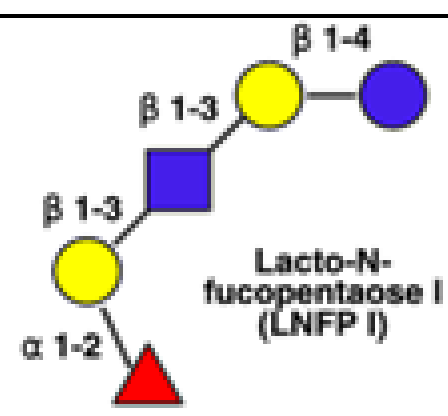	853.31	876.30
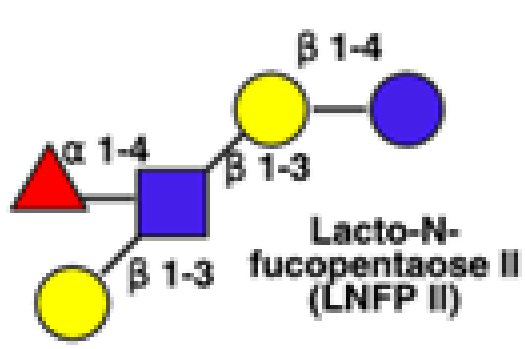	853.31	876.30
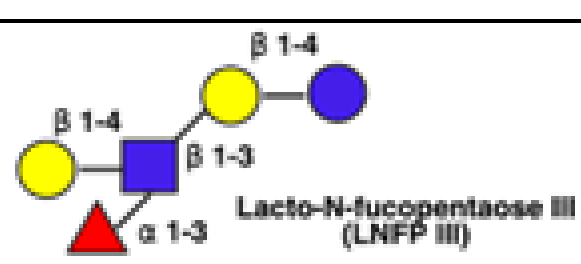	853.31	876.30

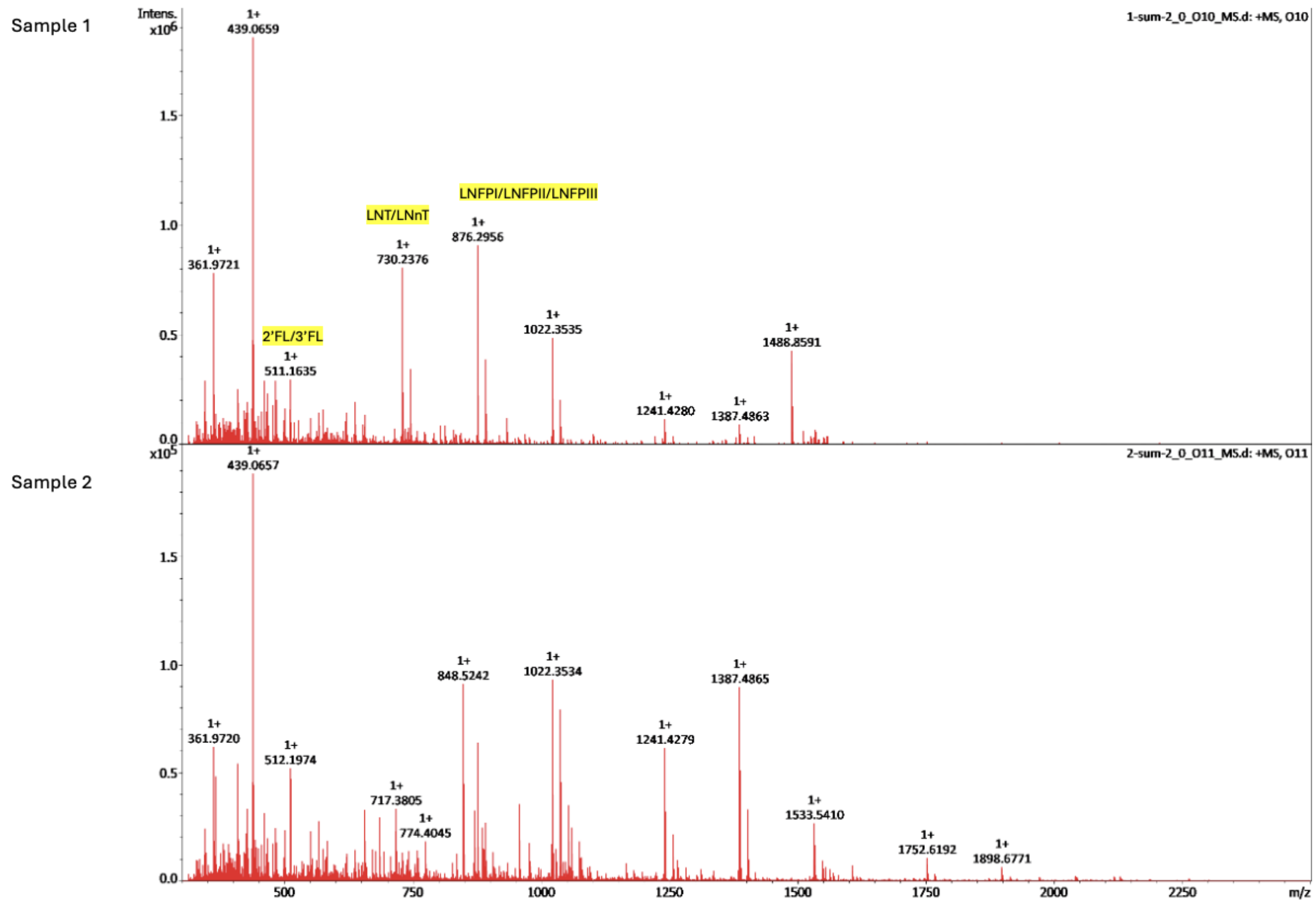

Figure S1. MALDI-tims-TOF MS/MS spectra for HMO samples from two donors used in the HMO cocktail for this work.

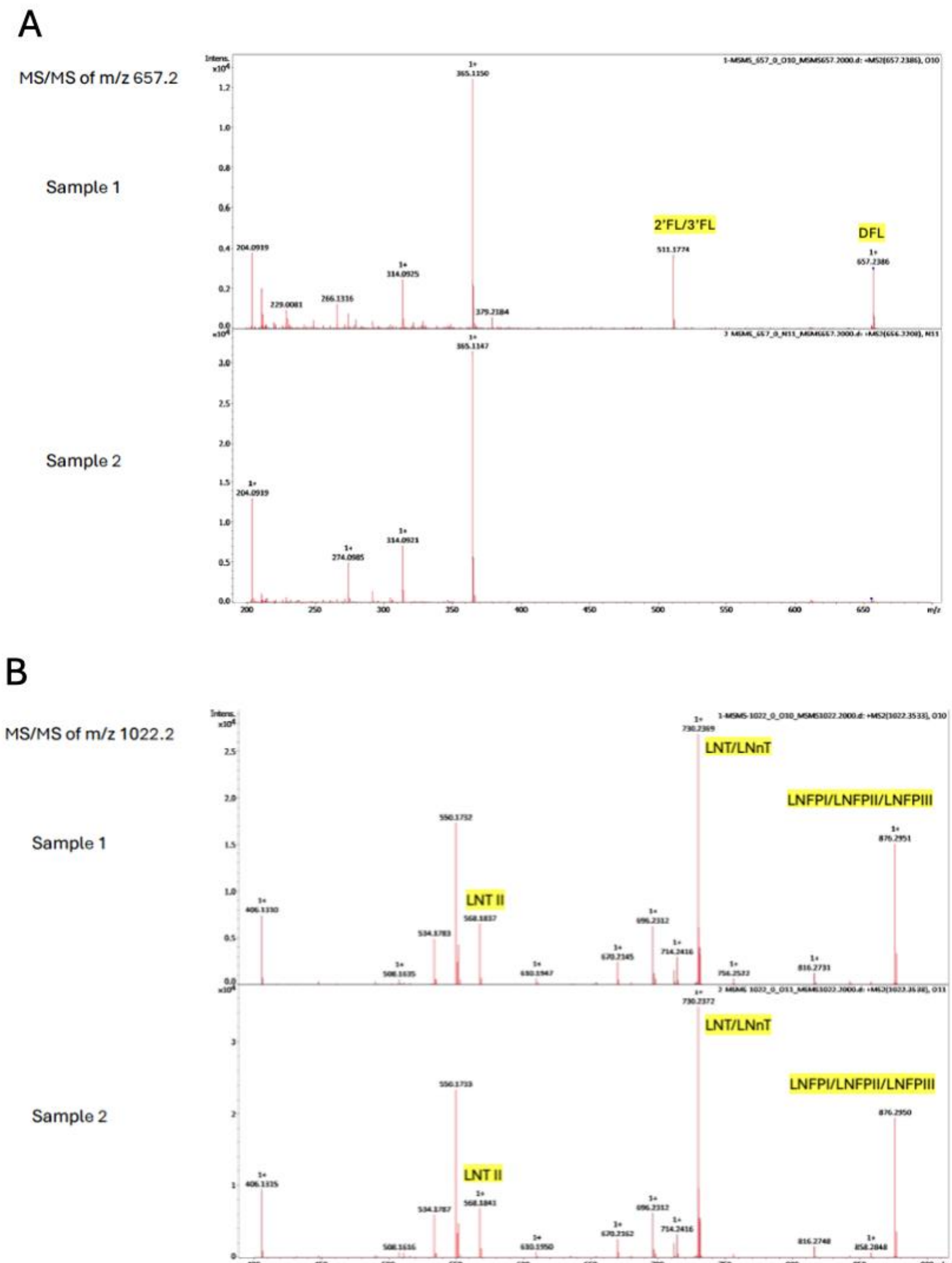

Figure S2. MS/MS of m/z 657.2 (A) and MS/MS of m/z 1022.2 (B) for HMO samples from two donors used in the HMO cocktail for this work.

Amperometry.

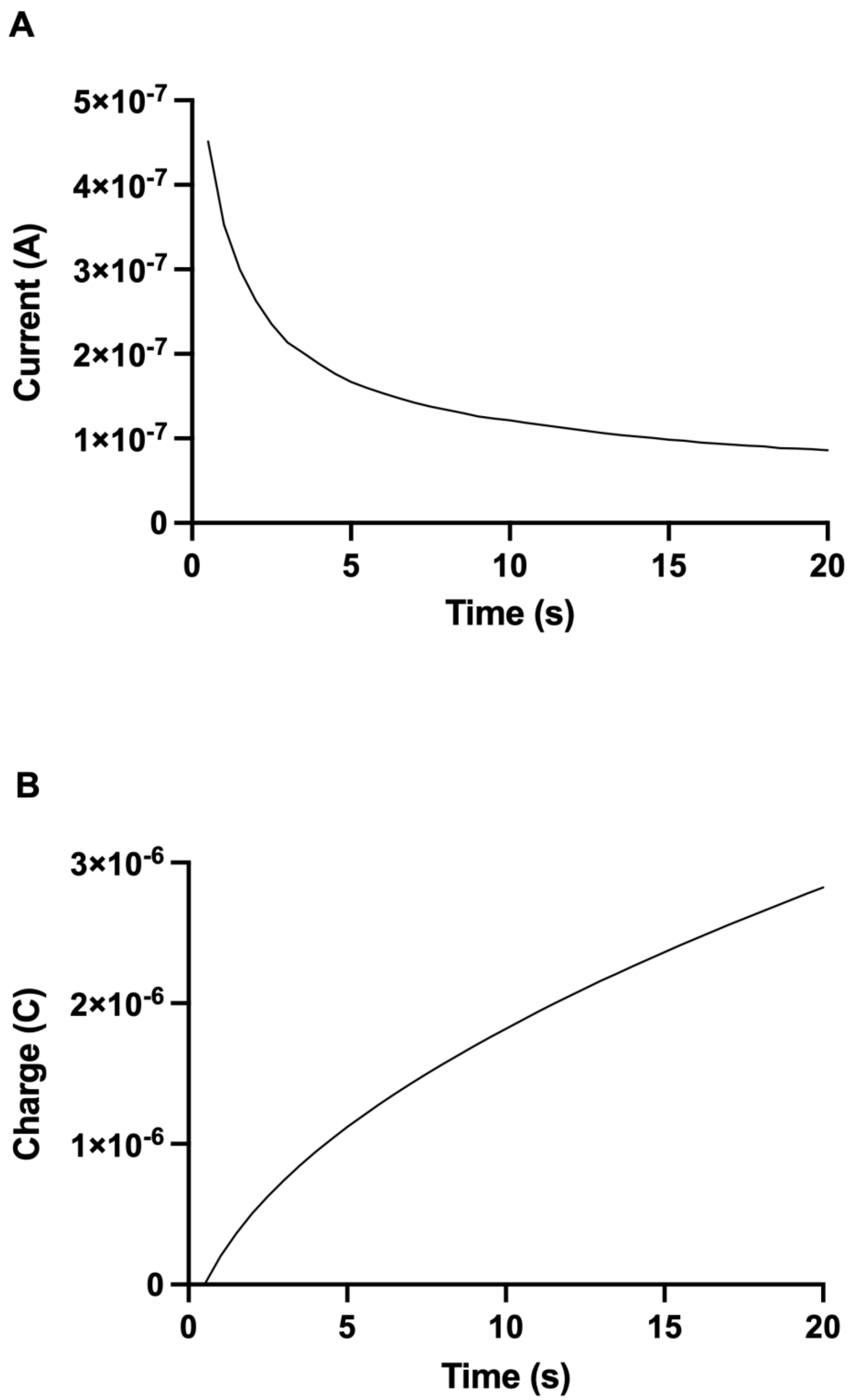

Figure S3. Amperometric (*i-t*) curve for 6000 pg/mL TNF-α (A) and integrated curve (B) used to obtain total charge for the system and generate Figure 2B.
